# Supplementary material for: Physical Gold Nanoparticle-Decorated Polyethylene Glycol-Hydroxyapatite Composites Guide Osteogenesis and Angiogenesis of Mesenchymal Stem Cells
Source: Biomedicines. 2021 Nov 6;9(11):1632. doi: 10.3390/biomedicines9111632 (PMC8615876; doi:10.3390/biomedicines9111632)
Supplement: Supplementary file 1 [file biomedicines-09-01632-s001.zip › biomedicines-1426973-supplementary.pdf]

biomedicines

Physical Gold Nanoparticle-Decorated Polyethylene Glycol-Hydroxyapatite Composites Guide Osteogenesis and Angiogenesis of Mesenchymal Stem Cells

Supplementary Data

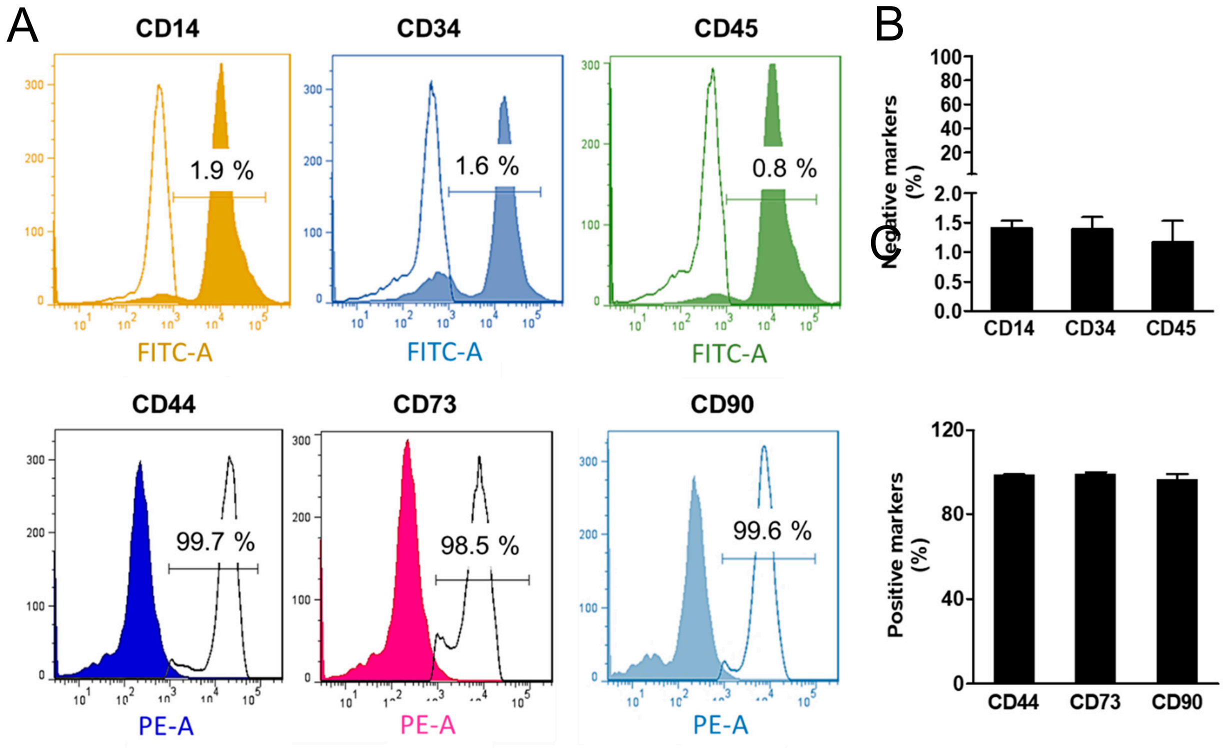

Figure S1. Characterization of MSCs using flow cytometry analysis. (A) Cells were harvested and incubated with the respective antibody conjugated with fluorescein isothiocyanate (FITC) and/or phycoerythrin (PE). The indicated markers: CD14-FITC, CD34-FITC, CD45-FITC, CD44-PE, CD73-PE, and CD90-PE (BD Pharmingen, USA). Filled area represents isotype controls. **(B)** The FACS results of negative markers: CD14, CD34, and CD45 expression. **(C)** The FACS data of positive markers: CD44, CD73, and CD90 expression.

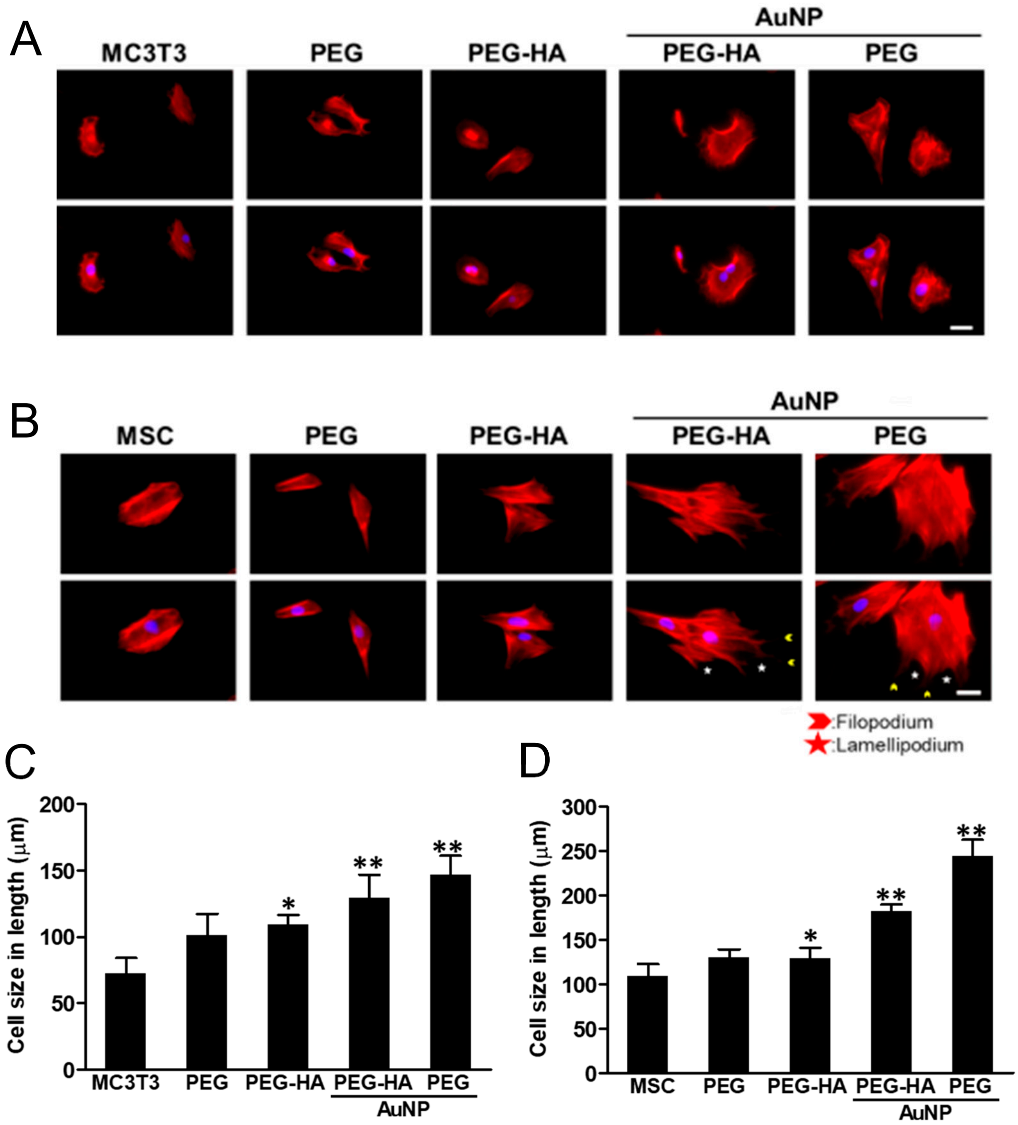

Figure S2. Cytoskeleton and cell morphology by rhodamine phalloidin staining of **(A)** MC3T3 cells and **(B)** MSCS for actin fiber extension on different materials after 8 h of incubation under fluorescence microscopy analysis. Arrows indicate filopodia (green color) and lamellipodia (red color). The actin fiber extension in length quantified by Image J software in **(C)** MC3T3 cells and **(D)** MSCs on different materials after 8 h is shown. Actin fiber length elongation was significantly observed in the PEG-HA-AuNP and PEG-AuNP test groups compared with the other groups. Data are mean ± SD (n = 3). *p < 0.05; **p < 0.05. Scale bar = 50 μm. Data are the mean ± SD (n = 3).

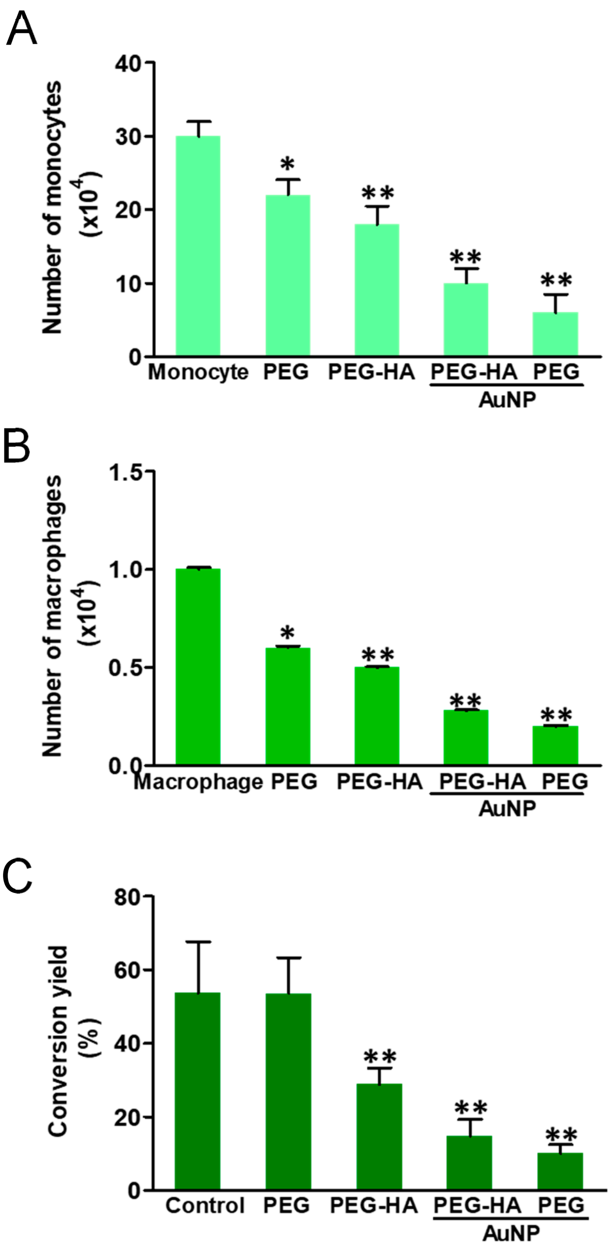

Figure S3. Quantification of the activation degree of monocytes and platelets. Number of **(A)** monocytes and **(B)** macrophages, and **(C)** conversion ratio of monocytes to macrophages. Data are the mean ± SD (n = 3). *$p<0.05$; **$p<0.01$: smaller than control (TCPS).

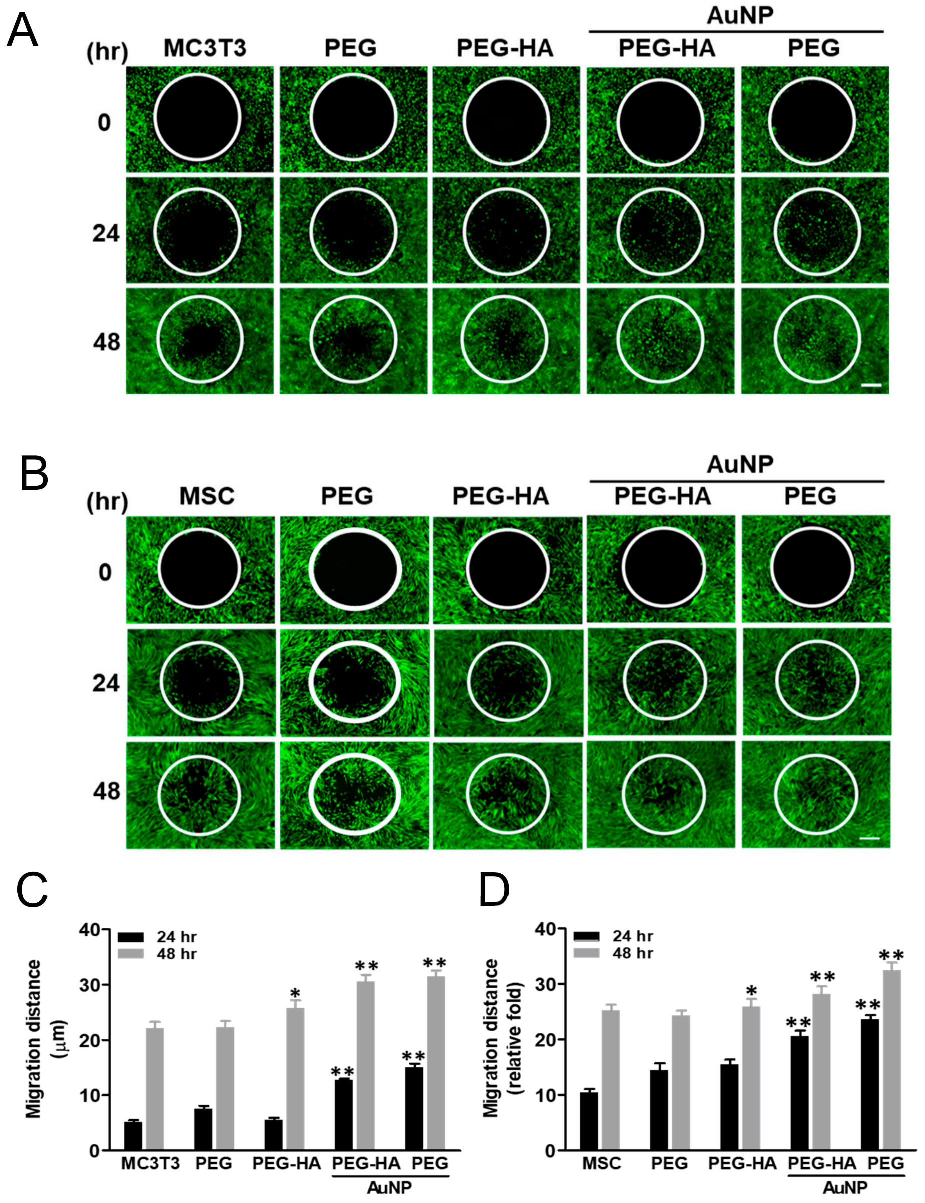

Figure S4. Migration of **(A)** MC3T3 cells and **(B)** MSCs on different materials during a period of 24 to 48 h. Cell migration into the gap zone area was monitored by fluorescence microscopy. Cells were stained by Calcein AM (2 μM) prior to examination. Scale bar = 500 μm. Quantification of the fluorescence intensity of **(C)** MC3T3 cells and **(D)** MSCs by Image J software. Data are the mean ± SD (n = 3). *p<0.05; **p<0.01: greater than control (TCPS). Data were obtained from three different experiments.

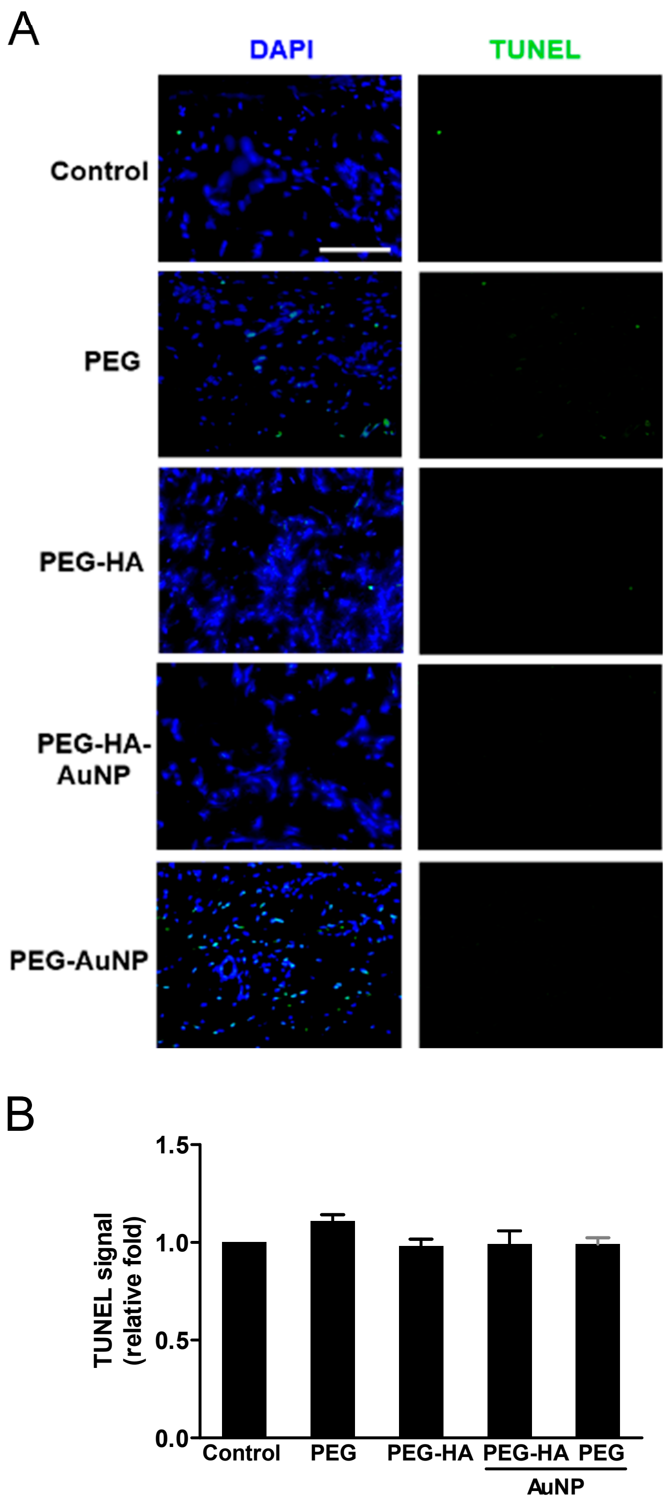

Figure S5. (A) Representative images of immunofluorescence staining for TUNEL-positive nuclei in different groups (20 × magnification). **(B)** Quantification of TUNEL-positive cells shows a lower number in the treated group as compared to the control group. Scale bar = 100 μm. The number of rats was 5 (n=5).

Table S1. XPS analysis of PEG and PEG composites

Sample	Atomic composition (%)					
	O_{1s}	C_{1s}	Ca_{2p}	P_{2p}	Au_{4f}	O_{1S}/C_{1S}
HA	34.69	58.88	6.43	-	-	0.59
PEG	63.92	36.08	-	-	-	1.77
PEG-AuNPs	54.61	45.39	-	-	a	1.20
PEG-HA	27.56	69.69	2.37	0.39	-	0.40
PEG-HA-AuNPs	50.13	37.62	4.56	7.7	a	1.33

[a] Since the AuNP peaks are not well defined, the percentage of Au 4f cannot be calculated.
